# Summer Diarrhœa in Children

**Published:** 1889-09

**Authors:** H. H. Thorpe

**Affiliations:** Liberty Hill, Texas


### SUMMER DIARRHŒA OF CHILDREN.

BY H. H. THORPE, M. D., LIBERTY HILL, TEXAS.

Read at Austin District Medical Society, and voted Published in Daniel's Texas Medical Journal.

IT BECOMES my duty to bring before you for discussion the disease known as the summer diarrhœa of children, more commonly called summer complaint, a disease which, in our larger cities, has been the annually recurring scourge of infancy, but which, thanks to sanitary regulations following the formation of boards of health, etc., is becoming less prevalent and less severe.

Situated as I am, in a country where the disease is usually of a mild type, and caused mainly by error of diet, I shall ask permission to cull from such literature as I have at command, in order to present the subject.

Two factors are recognized in this disease: atmospheric and dietetic.

The prevalence and severity corresponds with atmospheric heat, it being more severe in the hotter months. While more deaths occur in the fall than in the spring months, as, for instance, in October than in May, they are of cases that sicken in the hotter months, and only succumb after the cooler weather comes on.

As to the actual causative influence in vitiated atmosphere, we are at present unable to determine, although bacteria have been found in the dejections, and also penetrating the mucous membrane, and a smart attack of diarrhœa has been produced by the administration of the artificial cultivations, which would seem to indicate that they at least play a part in the causation. We might mention here that Henock, of Berlin, calls attention to intestinal mycosis. He claims that a fungus of diseased meat, some portion of which not being digested, settles upon intestinal surface and produces its effects, prominent among which is a severe diarrhœa.

The mode of feeding is a very important factor in the production of the diarrhœa, and doubtless a large proportion of those who fall victims to the disease would escape its consequences if feeding were properly adapted to the wants and digestive power of the infant. The plan of bottle feeding, and the too early resort to food prepared for older persons, alike produce an obstinate diarrhœa, which can only be controlled by conforming more closely to the demands of nature; also ill health or veneral excesses of the mother, or wet nurse, are undoubted causes. It is important that the nursing woman should lead a quiet and regular life as regards habit and diet.

Beginning usually with feverish fretfulness, the diarrhœa sets in, at first mild, with possibly greenish, acrid stools, containing undigested casein, sometimes accompanied by vomiting; in other cases the vomiting sets in later; the pulse accelerated according to the severity of the attack; the surface heat usually increased, though slightly in the milder cases; when from the continued diarrhœa the vital powers begin to fail, the surface temperature is lowered, the skin usually dry, the urinary secretions diminished, in some cases a dry cough is developed; in the latter stages the pupils become less sensitive to light, and are usually contracted.

As to treatment, we have the usual arrangement into preventive and curative measures.

As preventive measures, we have careful hygienic surroundings, with proper feeding as to kind of food, amount of food and regularity of feeding, all of which are important.

The infant should not be weaned during the hot weather.

The indications for treatment are, to provide proper food and pure air, to aid the digestive functions, to check the diarrhœa, and cure the intestinal catarrh.

The first and only satisfactory food is the secretion from the healthy breast. That unattainable, a selection must be made that conforms more nearly to the mother's milk, and possibly the plan proposed by Pfeiffer, of Weisbaden, is best, namely, peptonizing cow's milk, which, however, quickly spoils, and should be prepared in small quantities, and frequently.

The second indication, that of pure air, would include cleanliness of the infant, the clothes, the bedding, and all surroundings, and, if possible, the removal of the infant from city to country, in which case it should not be returned while the hot weather lasts.

To aid the digestive functions, some of the preparations of pepsin would be indicated, while to control the diarrhœa and cure the intestinal catarrh, after, where necessary, clearing out the alimentary passages with some mild purgative, as castor oil, opiates would be called for, combined with bismuth subnitrate, the opiate to be used with care where cerebral symptoms present themselves; or sometimes benefit would be had from the use of mercuric submuriate in small doses, combined with sugar of milk, chalk or bismuth.

Or, if the theory of micro-organism be accepted, an antiseptic treatment would be indicated, and possibly among the best remedies benzoate, or salicylate of sodium, or naphthalin, or resorcin, might be used.

Of course some cases would require moderate stimulation with an appropriate form of alcohol. Dr. Richard Pott and Dr. Diderich, of Halle, have reported favorably the use of a tincture of coca in infantile cholera, even when collapse was imminent. After an energetic use of the tincture for twelve to twenty-four hours, improvement set in.

